# Combined radiomics-clinical model to predict platinum-sensitivity in advanced high-grade serous ovarian carcinoma using multimodal MRI

**DOI:** 10.3389/fonc.2024.1341228

**Published:** 2024-01-24

**Authors:** Inye Na, Joseph J. Noh, Chan Kyo Kim, Jeong-Won Lee, Hyunjin Park

**Affiliations:** ^1^ Department of Electrical and Computer Engineering, Sungkyunkwan University, Suwon, Republic of Korea; ^2^ Gynecologic Cancer Center, Department of Obstetrics and Gynecology, Samsung Medical Center, Sungkyunkwan University School of Medicine, Seoul, Republic of Korea; ^3^ Department of Radiology and Center for Imaging Science, Samsung Medical Center, Sungkyunkwan University School of Medicine, Seoul, Republic of Korea; ^4^ Center for Neuroscience Imaging Research, Institute for Basic Science, Suwon, Republic of Korea

**Keywords:** ovarian high-grade serous carcinoma, platinum sensitivity, radiomics, machine learning, magnetic resonance imaging

## Abstract

**Introduction:**

We aimed to predict platinum sensitivity using routine baseline multimodal magnetic resonance imaging (MRI) and established clinical data in a radiomics framework.

**Methods:**

We evaluated 96 patients with ovarian cancer who underwent multimodal MRI and routine laboratory tests between January 2016 and December 2020. The patients underwent diffusion-weighted, contrast-enhanced T1-weighted, and T2-weighted MRI. Subsequently, 293 radiomic features were extracted by manually identifying tumor regions of interest. The features were subjected to the least absolute shrinkage and selection operators, leaving only a few selected features. We built the first prediction model with a tree-based classifier using selected radiomics features. A second prediction model was built by combining the selected radiomic features with four established clinical factors: age, disease stage, initial tumor marker level, and treatment course. Both models were built and tested using a five-fold cross-validation.

**Results:**

Our radiomics model predicted platinum sensitivity with an AUC of 0.65 using a few radiomics features related to heterogeneity. The second combined model had an AUC of 0.77, confirming the incremental benefits of the radiomics model in addition to models using established clinical factors.

**Conclusion:**

Our combined radiomics-clinical data model was effective in predicting platinum sensitivity in patients with advanced ovarian cancer.

## Introduction

Ovarian cancer is referred to as a ‘silent killer,’ due to its limited symptoms during the early stages. Therefore, 70% of cases are diagnosed at advanced stages (i.e., stages III/IV) resulting in a survival rate of less than 50% five years after the initial diagnosis (1–3). A combination of surgery and chemotherapy is recommended, and the extent of surgery varies widely according to individuals’ disease volume, from simple hysterectomy with bilateral salpingo-oophorectomy to multiple intestinal surgical procedures (4). Platinum-based chemotherapy is the standard first-line treatment option, and patients who relapse within 6 months of the end of first-line treatment are classified as ‘platinum-resistant’ and other patients as ‘platinum-sensitive’ (5). Approximately 25% of patients are platinum-resistant (6). These two types of patients undergo different subsequent treatment options; thus, it is important to distinguish between them as early as possible (5–7). Monitoring the response to platinum-based treatment with a change in tumor size is feasible, but requires significant manual effort. In addition, the treatment of recurrent ovarian cancer is more difficult, so delaying the first recurrence as much as possible is crucial, especially in advanced-stage patients (8, 9). Treatment options of recurrent disease should be individualized but generally include systemic therapy, secondary cytoreduction and radiotherapy (10). Thus, an efficient method, possibly one using machine learning, is required to predict platinum sensitivity.

Previous studies have investigated various factors to predict platinum sensitivity, including histological subtypes, BRCA1/2 mutations, homologous recombination deficiency (HRD), and further subclassifications based on genomic expression profiles (11–15). Numerous studies have demonstrated that germline BRCA1/2 mutations positively impact the overall survival and platinum response (16). Pennington et al. showed that the presence of germline and somatic homologous recombination mutations was highly predictive of primary platinum sensitivity (17). In a blood-based study, Matte et al. investigated the differences in cancer antigen 125 (CA125) and leptin levels in preoperative serum and intraoperative ascites between platinum-sensitive and platinum-resistant patients. Their results suggested that the serum CA125 to ascites leptin ratio is a novel biomarker for poor outcomes in patients with platinum-resistant high-grade serous carcinoma (HGSC) (18).

Magnetic resonance imaging (MRI) is a useful diagnostic modality in epithelial ovarian cancer. Diffusion-weighted imaging (DWI) is particularly helpful in assessing operability in this disease type (19, 20). DWI has high sensitivity for distinguishing between benign and malignant tumors based on their shape and texture information (21). However, previous studies using MRI-based radiomics have predominantly focused on improving precision diagnostics and the classification of histologic subtypes, and only a few studies have explored the utility of this imaging tool in developing models for predicting platinum sensitivity (22–24). In this study, we used multimodal MRI to comprehensively assess ovarian-cancer-related information, specifically focusing on platinum sensitivity.

Radiomics is a non-invasive method for extracting and analyzing high-dimensional quantifiable imaging features from routine medical imaging (25). Numerous studies have utilized this method for cancer analysis within machine learning frameworks (26–28). This method can evaluate tumor heterogeneity through shape and texture features and has been extensively used as an imaging-based biomarker for diagnosis, prognosis, and response assessment (29–31).

The purpose of this study was to evaluate whether a machine-learning model combining radiomics features derived from multimodal MRI and known clinical factors (e.g., age and disease stage) available at baseline can predict platinum response in patients with advanced-stage ovarian HGSC.

## Materials and methods

### Patient selection and clinicopathogical parameters

This retrospective study was approved by the Institutional Review Board, and the requirement for informed consent was waived. The study population (n = 100) was selected from patients diagnosed with ovarian HGSC at a tertiary academic medical center (Samsung Medical Center in Seoul, South Korea) between January 2016 and December 2020. The inclusion criteria were pretreatment pelvic MRI, histologically confirmed HGSC of the ovary, International Federation of Gynecology and Obstetrics (FIGO) stage IIIC – IVB, standard treatment with primary debulking surgery (PDS) followed by first-line platinum-based chemotherapy or neoadjuvant platinum-based chemotherapy followed by interval debulking surgery (IDS), and available follow-up records after chemotherapy for at least 6 months. Exclusion criteria were absence of clinical data, poor imaging quality, and incomplete chemotherapy treatment. The clinical factors analyzed included age at diagnosis, initial CA125 levels, tumor differentiation grades classified by the FIGO system (grades 2 and 3), extent of disease status classified by the FIGO system (FIGO 2014 stage IIIC through IVB), and residual disease after PDS or IDS. We analyzed 96 patients after applying the exclusion criteria.

### MRI acquisition protocols and tumor region of interest

**Figure 1:**
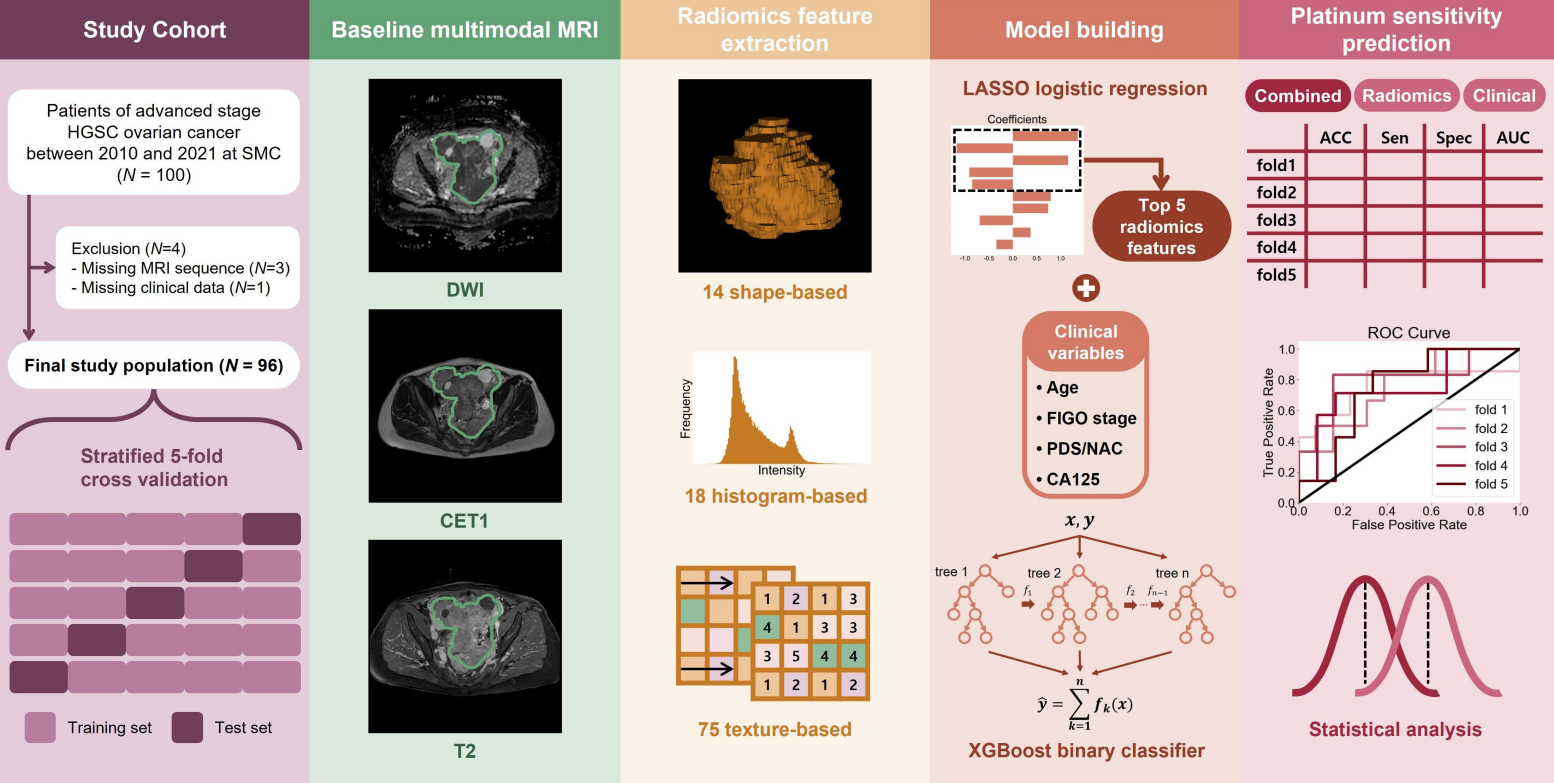
Overall procedures of the study.

**Figure 2:**
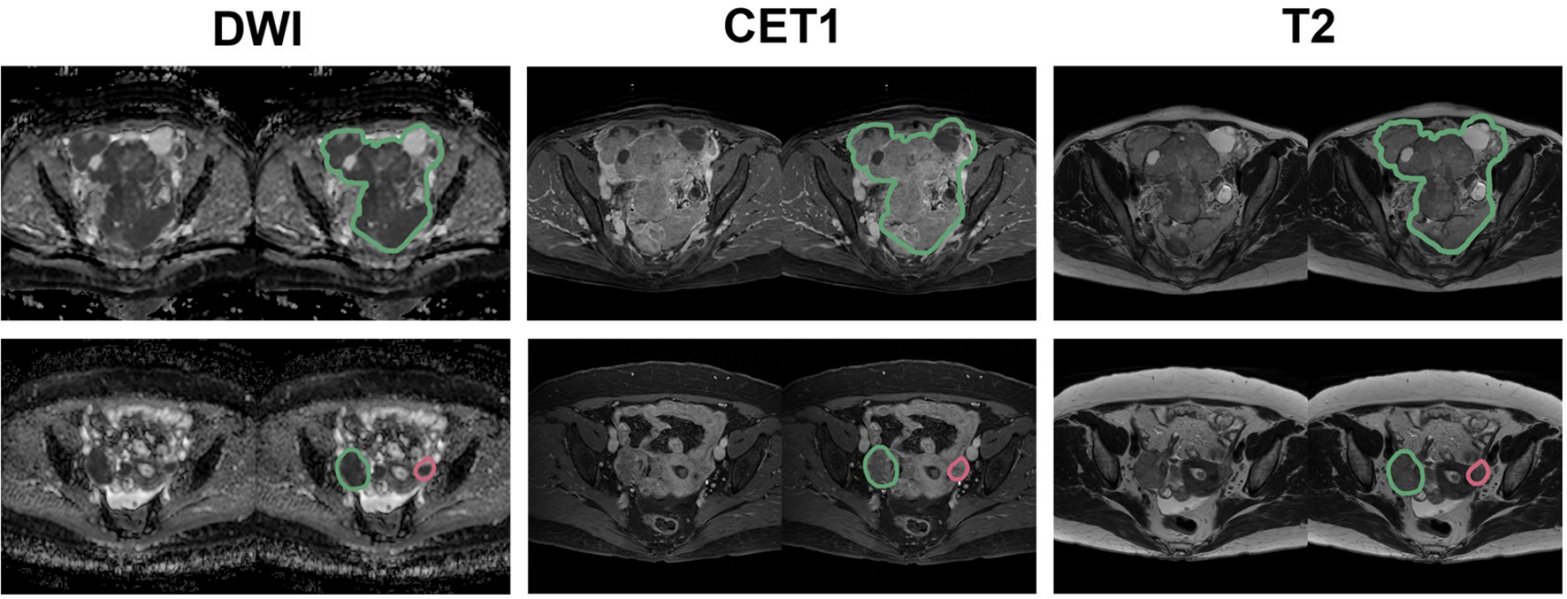
Representative placement of ROIs. The green contour indicates the largest tumor, while the red contour indicates the second-largest tumor for DWI, CET1, and T2 images.

All patients underwent pelvic MRI before treatment. In the present institution, computed tomography (CT) and magnetic resonance (MR) images are taken in all patients suspected to have ovarian malignancies for detailed characterization of adnexal masses. Axial T2-weight images (T2WI), fat-suppressed contrast-enhanced T1-weighted images (CE-T1WI), and DWI were used for the analysis (**Figure 1**). A genitourinary radiologist with 15 years of experience in interpreting female pelvic MRI was blinded to the patients’ clinical data and follow-up results and manually placed the region of interest (ROI) along the boundary of the primary tumor layer-by-layer to include the whole volume (cystic and solid components) on T2WI and CE-T1WI. The procedure was performed on each axial slice of the tumor using the 3D Slicer software (version 5.2.2). The tumor ROI measurements encompassed the maximum possible lesion extent in the image with the greatest visibility, as shown in **Figure 2**. For patients with multiple tumors, we identified the two largest tumors. The ROIs defined on DWI were transferred to T2WI and CE-T1WI with rigid image registration. All MRI images were voxel space-resampled with a spacing of 1 × 1 × 5 mm^3^ and interpolated using a B-spline.

### Radiomics features extraction and preprocessing

Radiomics features were extracted from each ROI of DWI, T2, and CET1 sequences using the open-source Python package “Pyradiomics” (version 3.0.1) (Python Software Foundation, Wilmington, DE, United States) (32). A total of 107 features were extracted, consisting of 14 shape-based features, 18 first-order statistical features, and 75 texture-based features (24 from the gray-level co-occurrence matrix, 16 from the gray-level size zone matrix, 16 from the gray-level run length matrix, 5 from the neighboring gray-tone difference matrix, and 14 from the gray-level dependence matrix) (**Supplementary Table 1**). Shape-based features were extracted from DWI alone, resulting in 293 features per patient. If a patient had multiple lesions, a weighted sum was performed based on the volume of the lesions, except for the “voxel volume” feature, which used the sum of the lesions. All radiomics features were z-score-normalized based on the mean and standard deviation of the training set. Details regarding the splitting of the data into training and test sets are provided below.

### Feature selection and model building

From the radiomics features, the top five features with the highest absolute value of the coefficient for each fold were selected using least absolute shrinkage and selection operator (LASSO) logistic regression with the target variable of initial platinum sensitivity. This reduces overfitting of the model. For comparison, we established two additional feature sets: one consisting of four clinical variables and the other combining five radiomic features with four clinical variables. The four clinical variables selected were patient age at diagnosis, disease stage, initial CA125 level, and whether the patient underwent PDS followed by platinum-based chemotherapy or platinum-based neoadjuvant chemotherapy followed by IDS. These four variables were selected because they are usually considered prognostic factors for the survival of women with advanced epithelial ovarian cancer (33, 34).

We built three machine learning models to predict initial platinum sensitivity using the XGBoost classifier, which sequentially trains and ensembles multiple tree-based classifiers. These are referred to as radiomic, clinical, and combined models. SHapley Additive exPlanations (SHAP) was adopted to explain the extent to which each feature in the models influenced the prediction, which allowed us to see a positive or negative correlation with platinum sensitivity.

### Statistical analysis

To compare the clinical characteristics between platinum-sensitive and platinum-resistant patients, Student’s t-test was used for continuous data (age, CA125), Mann–Whitney U test was used for ordinal data (grade, FIGO stage, residual disease), chi-square test was used for nominal data (PDS/neoadjuvant chemotherapy[NAC]), and log-rank test was used for time-to-event data (recurrence-free survival and overall survival). Survival analysis was performed by estimating Kaplan–Meier survival curves for recurrence and survival. Continuous data were presented as mean and standard deviation (SD) or median and interquartile range (IQR), and categorical data (nominal and ordinal data) were presented as numbers of values and percentages.

To evaluate the generalization performance of the models, we performed 5-fold cross-validation. The 96 patients were divided into five groups while maintaining the ratio of sensitive/resistant patients, using 4 folds as the training set and the remaining fold as the test set. The procedure was repeated five times, using a different fold as the test set. We performed data preprocessing, feature selection, model training with the training set, and model evaluation with the test set for a total of 5 times resulting in 5 models built. To evaluate the performance of the model in classifying platinum sensitivity, we calculated the accuracy, specificity, sensitivity, and area under the curve (AUC) of the receiver operating characteristic curves. The thresholds for accuracy, specificity, and sensitivity were set to 0.5. Finally, the mean and SD were presented together to comprehensively evaluate the performance of the models over five folds.

## Results

The clinical characteristics of the platinum-sensitive and platinum-resistant patients are compared in **Table 1**. Univariate analysis revealed that no clinical factors were significantly associated with platinum sensitivity (age, tumor differentiation grade, disease stage, CA125, PDS/NAC, or residual tumor after surgery; all p-values > 0.05). However, the patients who relapsed within 6 months of the last administration of a platinum-based chemotherapy demonstrated significantly shorter overall survival in comparison to those who relapsed 6 months after the last platinum-based chemotherapy (13.3 months vs. 53.1 months in platinum-resistant patients vs. platinum-sensitive patients; p-value < 0.001) (**Table 1** and **Supplementary Figure 1**).

**Table 1:** Characteristics of patients. Various clinical parameters are presented for the platinum-sensitive and -resistant groups.

Clinical parameters	All (*N=96*)	Platinum-Sensitive (*N=63, 65.6%*)	Platinum-Resistant (*N=33, 34.4%*)	*p-value*
**Follow-up duration, months, median (IQR)**	45.29 (25.96-59.63)	53.03 (41.81-71.61)	18.83 (14.92-28.81)	NA
**Age, years, mean ± SD**	57.15 ± 10.58	55.83 ± 11.14	59.67 ± 9.05	.09^a^
**Grade, *N* (%)**				.09^b^
2	10 (10.4)	9 (14.3)	1 (3.0)	
3	86 (89.6)	54 (85.7)	32 (97.0)	
**FIGO Stage 2014, *N* (%)**				.06^b^
IIIC	69 (71.9)	49 (77.8)	20 (60.6)	
IVA	2 (2.1)	2 (3.2)	0 (0)	
IVB	25 (26.0)	12 (19.0)	13 (39.4)	
**CA-125 baseline, mean ± SD**	2001.30 ± 2752.83	2124.76 ± 3197.42	1765.62 ± 1613.11	.47^a^
**Presence of ascites before treatment (%)**	75 (78)	48 (76)	27 (82)	.53^c^
**Germline BRCA mutation**				.44^c^
Wildtype	79	51	30	
BRCA1 mutant	11	8	2	
BRCA2 mutant	6	4	1	
**Primary treatment strategy, N (%)**				.66^c^
PDS	71 (74.0)	48 (76.2)	23 (69.7)	
NAC	25 (26.0)	15 (23.8)	10 (30.3)	
**Residual disease, *N* (%)**				.11^b^
No	34 (35.4)	25 (39.7)	9 (27.3)	
< 5mm	22 (22.9)	15 (23.8)	7 (21.2)	
5mm - 1cm	11 (11.5)	7 (11.1)	4 (12.1)	
1cm - 2cm	6 (6.2)	4 (6.3)	2 (6.1)	
> 2cm	23 (24.0)	12 (19.0)	11 (33.3)	
**Recurrence, *N* (%)**	89 (92.7)	56 (88.9)	33 (100)	<0.001^d^
Recurrence-free survival, months, median (IQR)	7.29 (4.21-11.93)	7.52 (6.21-13.68)	3.94 (2.79-4.67)	
**Death, *N* (%)**	40 (41.7)	23 (36.5)	17 (51.5)	<0.001^d^
Overall-survival, months, median (IQR)	33.50 (14.44-54.81)	53.06 (36.52-71.69)	13.27 (9.50-19.65)	

^a^Student’s t-test, ^b^Mann-Whitney U test, ^c^Chi-square test, ^d^Log-rank test.

**Table 2** shows the frequently selected features more than one-fold as a result of the LASSO feature selection. The texture-based feature of small dependence low grey level emphasis (SDLGLE) extracted from the gray level dependence matrix (GLDM) of CE-T1WI was selected in all five folds.

**Table 2:** Frequently selected radiomics features over 5 folds.

Modality	Category	Feature	Count
CE-T1	Texture GLDM^a^	Small Dependence Low Gray Level Emphasis	5
DWI	Texture NGTDM^a^	Busyness	4
DWI	Shape	Flatness	3
T2	Histogram	10 Percentile	2
DWI	Shape	Sphericity	2
T2	Histogram	Interquartile Range	2

^a^GLDM, Gray Level Dependence Matrix; NGTDM, Neighbouring Gray-Tone Difference Matrix.

Each fold-specific model was trained with nine features, combining four clinical variables and five radiomics features. **Supplementary Figure 2** shows the SHAP values of the training set for each feature of the trained XGBoost classifier. For example, for the FIGO stage, there are many pink dots where the SHAP value is negative. This can be interpreted as the FIGO stage negatively correlating with platinum sensitivity. Conversely, if there were more pink dots where the SHAP value was positive, the features were positively correlated. Among the top three most frequently selected radiomic features, the SDLGLE feature calculated from the GLDM of CET1-MRI was negatively correlated with platinum sensitivity, busyness features calculated from the NGTDM of DWI were positively correlated, and flatness was negatively correlated.

**Table 3** summarizes the performance of the test set for each fold of the radiomic, clinical, and combined models to classify platinum sensitivity and platinum resistance. The combined model, which combines radiomics features and clinical variables, showed the best classification performance with an average accuracy of 0.71 and an AUC of 0.77.

**Table 3:** Classification performance of radiomics, clinical, and combined models over 5 folds to distinguish between the platinum-sensitive and -resistant groups.

Radiomics	Accuracy	Sensitivity	Specificity	AUC
fold 1	0.8000	0.8571	0.7692	0.8242
fold 2	0.5263	0.1667	0.6923	0.5769
fold 3	0.7895	0.5000	0.9231	0.7436
fold 4	0.6842	0.4286	0.8333	0.5952
fold 5	0.5789	0.2857	0.7500	0.5000
**Mean ± SD**	**0.6758 ± 0.1097**	**0.4476 ± 0.2349**	**0.7936 ± 0.0789**	**0.648 ± 0.1182**

Clinical	Accuracy	Sensitivity	Specificity	AUC
fold 1	0.7	0.5714	0.7692	0.6484
fold 2	0.6316	0.6667	0.6154	0.8077
fold 3	0.7368	0.3333	0.9231	0.6538
fold 4	0.6316	0.4286	0.75	0.5952
fold 5	0.7895	0.4286	1.0	0.7262
**Mean ± SD**	**0.6979 ± 0.0612**	**0.4857 ± 0.1182**	**0.8115 ± 0.1357**	**0.6863 ± 0.0736**

Combined	Accuracy	Sensitivity	Specificity	AUC
fold 1	0.75	0.5714	0.8462	0.7692
fold 2	0.6316	0.6667	0.6154	0.7564
fold 3	0.7368	0.3333	0.9231	0.8077
fold 4	0.7895	0.7143	0.8333	0.75
fold 5	0.6316	0.4286	0.75	0.75
**Mean ± SD**	**0.7079 ± 0.0647**	**0.5429 ± 0.1432**	**0.7936 ± 0.1047**	**0.7667 ± 0.0217**

## Discussion

The present study investigated the role of a combined radiomics-clinical data model in predicting platinum sensitivity in patients with advanced ovarian HGSC. The combined model, which was developed by integrating MRI radiomic features and clinical data, performed better than the MRI model or the clinical model alone in predicting platinum sensitivity.

Platinum-based chemotherapy is the first-line treatment for advanced ovarian HGSC. However, identifying patients who are likely to demonstrate a treatment response to primary platinum-based chemotherapy is challenging. Therefore, it is essential to search for adequate predictive markers that can be easily performed in clinical practice to identify patients who can maximally benefit from treatment. Previous studies have demonstrated that radiomic information from MRI can improve the efficiency of precise diagnosis by leveraging high-resolution morphological images and providing various functional information, such as tissue oxygenation, perfusion, or diffusion (35–38). In addition, recent studies have demonstrated that radiomics information from MRI can be used to predict the treatment response to platinum, risk of recurrence, and residual disease in patients with ovarian HGSC (39–41). By combining clinical predictive markers with radiomics information extracted from pretreatment MRI data, the present study successfully demonstrated the enhanced ability of the model to predict platinum sensitivity in women with advanced ovarian HGSC.

Based on frequently selected radiomics features, we can gain insight into how each radiomics feature is associated with platinum sensitivity. First, the SDLGLE of the GLDM is high when similar patterns of low-intensity regions occur nearby. This suggests that the uniformly textured low-intensity regions of a tumor appearing on CET1-MRI are related to platinum sensitivity. Second, the busyness value of the NGTDM is high when the intensity difference between neighboring pixels is large. This suggests that the intensity of the tumor region on DWI-MRI is more platinum-sensitive when it appears as a high-contrast and varied texture. Finally, flatness was higher when the region of interest was flat and thin. Our results suggest that tumors with plate-like structures are platinum-resistant, whereas those with spherical or cylindrical structures are platinum-sensitive. The first two features are related to the intensity/texture heterogeneity within the ROI. Similar to other tumors, heterogeneity may play an important role in advanced HGSC.

Considerable efforts have been devoted to recent studies to understanding the possible mechanisms of platinum resistance along with poly ADP ribose polymerase (PARP) inhibitor resistance (42). Most patients who relapse with a progression-free interval of less than six months after platinum-based chemotherapy exhibit little to no response to other agents. Consequently, survival rates have not significantly improved for advanced-stage ovarian cancer over the last several decades, with a 5-year survival rate of 20 – 27% (34, 43, 44). Driven by advances in the molecular and genomic understanding of epithelial ovarian cancer, researchers are slowly gaining insight into the potential mechanisms by which platinum resistance develops in this patient population. However, our current understanding does not provide a clear view of platinum sensitivity, and much remains to be explored. Substantial advances in imaging techniques and their applications have been achieved in recent years. Computational analysis techniques that combine radiomics information and clinical data that are already known to be associated with survival or treatment response may enhance our ability to predict platinum sensitivity. The potential role of radiomics information should be further explored in relation to other already-known survival predictors such as platinum sensitivity and tumor resectability. Rigorous technical, biological, and clinical validation of this rapidly emerging field of imaging research is required for clinical applications.

Our study has several limitations. First, this was a single-institution retrospective study. Thus, our findings need to be validated in a multi-institution prospective setting. Second, machine-learning studies are increasingly adopting deep-learning methods; however, such methods require larger samples. Therefore, such investigations are left for future research. Third, establishing a direct link between radiomics features and the molecular mechanism of HGSC requires a rich array of genomic data, including BRCA mutations and HRD. This study did not examine these parameters, which we plan to investigate in future studies.

We demonstrated the effectiveness of our combined radiomics-clinical data model in predicting platinum sensitivity in patients with advanced ovarian HGSC. Our results may contribute to enhanced personalized treatment of women with advanced ovarian HGSC.

## Data availability statement

The raw data supporting the conclusions of this article will be made available by the authors, without undue reservation.

## Ethics statement

The studies involving humans were approved by Samsung Medical Center Institutional Review Board. The studies were conducted in accordance with the local legislation and institutional requirements. Written informed consent for participation was not required from the participants or the participants’ legal guardians/next of kin in accordance with the national legislation and institutional requirements.

## Author contributions

IN: Writing – original draft. JN: Writing – original draft. CK: Formal analysis, Writing – review & editing. JL: Conceptualization, Writing – review & editing. HP: Conceptualization, Writing – review & editing.

## References

[1] Matulonis UA Sood AK Fallowfield L Howitt BE Sehouli J Karlan BY. Ovarian cancer. Nat Rev Dis Primers (2016) 2:16061. doi: 10.1038/nrdp.2016.61 27558151 PMC7290868

[2] Bankhead CR Collins C Stokes-Lampard H Rose P Wilson S Clements A. Identifying symptoms of ovarian cancer: a qualitative and quantitative study. BJOG (2008) 115(8):1008–14. doi: 10.1111/j.1471-0528.2008.01772.x PMC2607526 18651882

[3] Millstein J Budden T Goode EL Anglesio MS Talhouk A Intermaggio MP. Prognostic gene expression signature for high-grade serous ovarian cancer. Ann Oncol (2020) 31(9):1240–50. doi: 10.1016/j.annonc.2020.05.019 PMC7484370 32473302

[4] Mereu L Dalpra F Berlanda V Pertile R Coser D Pecorino B. Anastomotic leakage after colorectal surgery in ovarian cancer: drainage, stoma utility and risk factors. Cancers (Basel) (2022) 14(24):6243. doi: 10.3390/cancers14246243 36551728 PMC9776666

[5] Davis A Tinker AV Friedlander M. "Platinum resistant" ovarian cancer: what is it, who to treat and how to measure benefit? Gynecol Oncol (2014) 133(3):624–31. doi: 10.1016/j.ygyno.2014.02.038 24607285

[6] Pignata S Ferrandina G Scarfone G Scollo P Odicino F Selvaggi L. Extending the platinum-free interval with a non-platinum therapy in platinum-sensitive recurrent ovarian cancer. Results from the SOCRATES Retrospective Study. Oncology (2006) 71(5-6):320–6. doi: 10.1159/000108592 17878745

[7] Pignata S Ferrandina G Scarfone G Scollo P Odicino F Cormio G. Poor outcome of elderly patients with platinum-sensitive recurrent ovarian cancer: results from the SOCRATES retrospective study. Crit Rev Oncol Hematol (2009) 71(3):233–41. doi: 10.1016/j.critrevonc.2008.12.010 19179095

[8] Pignata S CC S Du Bois A Harter P Heitz F. Treatment of recurrent ovarian cancer. Ann Oncol (2017) 28(suppl_8):viii51–viii6. doi: 10.1093/annonc/mdx441 29232464

[9] Hoppenot C Eckert MA Tienda SM Lengyel E. Who are the long-term survivors of high grade serous ovarian cancer? Gynecol Oncol (2018) 148(1):204–12. doi: 10.1016/j.ygyno.2017.10.032 29128106

[10] Scandurra G Gieri S Marletta F Pecorino B Nicolini S Banna GL. Safety and efficacy of new techniques of radiotherapy in oligometastatic recurrent ovarian cancer patients with BRCA1/2 mutation. Eur J Gynaecol Oncol (2019) 40(5):739–43. doi: 10.12892/ejgo4639.2019

[11] Alkema NG Wisman GB van der Zee AG van Vugt MA de Jong S. Studying platinum sensitivity and resistance in high-grade serous ovarian cancer: Different models for different questions. Drug Resist Updat (2016) 24:55–69. doi: 10.1016/j.drup.2015.11.005 26830315

[12] Zhong Q Peng HL Zhao X Zhang L Hwang WT. Effects of BRCA1- and BRCA2-related mutations on ovarian and breast cancer survival: a meta-analysis. Clin Cancer Res (2015) 21(1):211–20. doi: 10.1158/1078-0432.CCR-14-1816 PMC4286460 25348513

[13] Itamochi H Kigawa J Terakawa N. Mechanisms of chemoresistance and poor prognosis in ovarian clear cell carcinoma. Cancer Sci (2008) 99(4):653–8. doi: 10.1111/j.1349-7006.2008.00747.x PMC11158134 18377417

[14] Pisano C Greggi S Tambaro R Losito S Iodice F Di Maio M. Activity of chemotherapy in mucinous epithelial ovarian cancer: a retrospective study. Anticancer Res (2005) 25(5):3501–5. 16101169

[15] Tothill RW Tinker AV George J Brown R Fox SB Lade S. Novel molecular subtypes of serous and endometrioid ovarian cancer linked to clinical outcome. Clin Cancer Res (2008) 14(16):5198–208. doi: 10.1158/1078-0432.CCR-08-0196 18698038

[16] Khan MA Vikramdeo KS Sudan SK Singh S Wilhite A Dasgupta S. Platinum-resistant ovarian cancer: From drug resistance mechanisms to liquid biopsy-based biomarkers for disease management. Semin Cancer Biol (2021) 77:99–109. doi: 10.1016/j.semcancer.2021.08.005 34418576 PMC8665066

[17] Pennington KP Walsh T Harrell MI Lee MK Pennil CC Rendi MH. Germline and somatic mutations in homologous recombination genes predict platinum response and survival in ovarian, fallopian tube, and peritoneal carcinomas. Clin Cancer Res (2014) 20(3):764–75. doi: 10.1158/1078-0432.CCR-13-2287 PMC3944197 24240112

[18] Matte I Garde-Granger P Bessette P Piche A. Serum CA125 and ascites leptin level ratio predicts baseline clinical resistance to first-line platinum-based treatment and poor prognosis in patients with high grade serous ovarian cancer. Am J Cancer Res (2019) 9(1):160–70. PMC6356915 30755819

[19] Michielsen K Dresen R Vanslembrouck R De Keyzer F Amant F Mussen E. Diagnostic value of whole body diffusion-weighted MRI compared to computed tomography for pre-operative assessment of patients suspected for ovarian cancer. Eur J Cancer (2017) 83:88–98. doi: 10.1016/j.ejca.2017.06.010 28734146

[20] De Piano F Buscarino V Maresca D Maisonneuve P Aletti G Lazzari R. Do DWI and quantitative DCE perfusion MR have a prognostic value in high-grade serous ovarian cancer? Radiol Med (2019) 124(12):1315–23. doi: 10.1007/s11547-019-01075-z 31473928

[21] Meng XF Zhu SC Sun SJ Guo JC Wang X. Diffusion weighted imaging for the differential diagnosis of benign vs. Malignant ovarian neoplasms. Oncol Lett (2016) 11(6):3795–802. doi: 10.3892/ol.2016.4445 PMC4888089 27313697

[22] Lei R Yu Y Li Q Yao Q Wang J Gao M. Deep learning magnetic resonance imaging predicts platinum sensitivity in patients with epithelial ovarian cancer. Front Oncol (2022) 12:895177. doi: 10.3389/fonc.2022.895177 36505880 PMC9727155

[23] Lu J Li HM Cai SQ Zhao SH Ma FH Li YA. Prediction of platinum-based chemotherapy response in advanced high-grade serous ovarian cancer: ADC histogram analysis of primary tumors. Acad Radiol (2021) 28(3):e77–85. doi: 10.1016/j.acra.2020.01.024 32061467

[24] Li H Cai S Deng L Xiao Z Guo Q Qiang J. Prediction of platinum resistance for advanced high-grade serous ovarian carcinoma using MRI-based radiomics nomogram. Eur Radiol (2023) 33(8):5298–308. doi: 10.1007/s00330-023-09552-w 36995415

[25] Lambin P Rios-Velazquez E Leijenaar R Carvalho S van Stiphout RG Granton P. Radiomics: extracting more information from medical images using advanced feature analysis. Eur J Cancer (2012) 48(4):441–6. doi: 10.1016/j.ejca.2011.11.036 PMC4533986 22257792

[26] Valdora F Houssami N Rossi F Calabrese M Tagliafico AS. Rapid review: radiomics and breast cancer. Breast Cancer Res Treat (2018) 169(2):217–29. doi: 10.1007/s10549-018-4675-4 29396665

[27] Cho HH Kim CK Park H. Overview of radiomics in prostate imaging and future directions. Br J Radiol (2022) 95(1131):20210539. doi: 10.1259/bjr.20210539 34797688 PMC8978251

[28] Nougaret S Tardieu M Vargas HA Reinhold C Vande Perre S Bonanno N. Ovarian cancer: An update on imaging in the era of radiomics. Diagn Interv Imaging (2019) 100(10):647–55. doi: 10.1016/j.diii.2018.11.007 30555018

[29] Nougaret S McCague C Tibermacine H Vargas HA Rizzo S Sala E. Radiomics and radiogenomics in ovarian cancer: a literature review. Abdom Radiol (NY) (2021) 46(6):2308–22. doi: 10.1007/s00261-020-02820-z 33174120

[30] Zhang H Mao Y Chen X Wu G Liu X Zhang P. Magnetic resonance imaging radiomics in categorizing ovarian masses and predicting clinical outcome: a preliminary study. Eur Radiol (2019) 29(7):3358–71. doi: 10.1007/s00330-019-06124-9 30963272

[31] Yao F Ding J Hu Z Cai M Liu J Huang X. Ultrasound-based radiomics score: a potential biomarker for the prediction of progression-free survival in ovarian epithelial cancer. Abdom Radiol (NY) (2021) 46(10):4936–45. doi: 10.1007/s00261-021-03163-z 34120235

[32] van Griethuysen JJM Fedorov A Parmar C Hosny A Aucoin N Narayan V. Computational radiomics system to decode the radiographic phenotype. Cancer Res (2017) 77(21):e104–e7. doi: 10.1158/0008-5472.CAN-17-0339 PMC5672828 29092951

[33] Lheureux S Braunstein M Oza AM. Epithelial ovarian cancer: Evolution of management in the era of precision medicine. CA Cancer J Clin (2019) 69(4):280–304. doi: 10.3322/caac.21559 31099893

[34] Lheureux S Gourley C Vergote I Oza AM. Epithelial ovarian cancer. Lancet (2019) 393(10177):1240–53. doi: 10.1016/S0140-6736(18)32552-2 30910306

[35] De Perrot T Sadjo Zoua C Glessgen CG Botsikas D Berchtold L Salomir R. Diffusion-weighted MRI in the genitourinary system. J Clin Med (2022) 11(7):1921. doi: 10.3390/jcm11071921 35407528 PMC9000195

[36] Song XL Ren JL Yao TY Zhao D Niu J. Radiomics based on multisequence magnetic resonance imaging for the preoperative prediction of peritoneal metastasis in ovarian cancer. Eur Radiol (2021) 31(11):8438–46. doi: 10.1007/s00330-021-08004-7 33948702

[37] Song XL Ren JL Zhao D Wang L Ren H Niu J. Radiomics derived from dynamic contrast-enhanced MRI pharmacokinetic protocol features: the value of precision diagnosis ovarian neoplasms. Eur Radiol (2021) 31(1):368–78. doi: 10.1007/s00330-020-07112-0 32767049

[38] Qian L Ren J Liu A Gao Y Hao F Zhao L. MR imaging of epithelial ovarian cancer: a combined model to predict histologic subtypes. Eur Radiol (2020) 30(11):5815–25. doi: 10.1007/s00330-020-06993-5 32535738

[39] Li HM Gong J Li RM Xiao ZB Qiang JW Peng WJ. Development of MRI-based radiomics model to predict the risk of recurrence in patients with advanced high-grade serous ovarian carcinoma. AJR Am J Roentgenol (2021) 217(3):664–75. doi: 10.2214/AJR.20.23195 34259544

[40] Li H Zhang R Li R Xia W Chen X Zhang J. Noninvasive prediction of residual disease for advanced high-grade serous ovarian carcinoma by MRI-based radiomic-clinical nomogram. Eur Radiol (2021) 31(10):7855–64. doi: 10.1007/s00330-021-07902-0 33864139

[41] Li C Wang H Chen Y Fang M Zhu C Gao Y. A nomogram combining MRI multisequence radiomics and clinical factors for predicting recurrence of high-grade serous ovarian carcinoma. J Oncol (2022) 2022:1716268. doi: 10.1155/2022/1716268 35571486 PMC9095390

[42] Li H Liu ZY Wu N Chen YC Cheng Q Wang J. PARP inhibitor resistance: the underlying mechanisms and clinical implications. Mol Cancer (2020) 19(1):107. doi: 10.1186/s12943-020-01227-0 32563252 PMC7305609

[43] Yang L Xie HJ Li YY Wang X Liu XX Mai J. Molecular mechanisms of platinum−based chemotherapy resistance in ovarian cancer (Review). Oncol Rep (2022) 47(4):82. doi: 10.3892/or.2022.8293 35211759 PMC8908330

[44] Ortiz M Wabel E Mitchell K Horibata S. Mechanisms of chemotherapy resistance in ovarian cancer. Cancer Drug Resist (2022) 5(2):304–16. doi: 10.20517/cdr.2021.147 PMC9255249 35800369

