# Supplementary material for: Combined radiomics-clinical model to predict platinum-sensitivity in advanced high-grade serous ovarian carcinoma using multimodal MRI
Source: Front Oncol. 2024 Jan 24;14:1341228. doi: 10.3389/fonc.2024.1341228 (PMC10847571; doi:10.3389/fonc.2024.1341228)
Supplement: Supplementary file 1 [file DataSheet_1.pdf]

Supplementary Figure 1. Kaplan–Meier plots of recurrence-free survival and overall survival among the platinum-sensitive and -resistant groups.

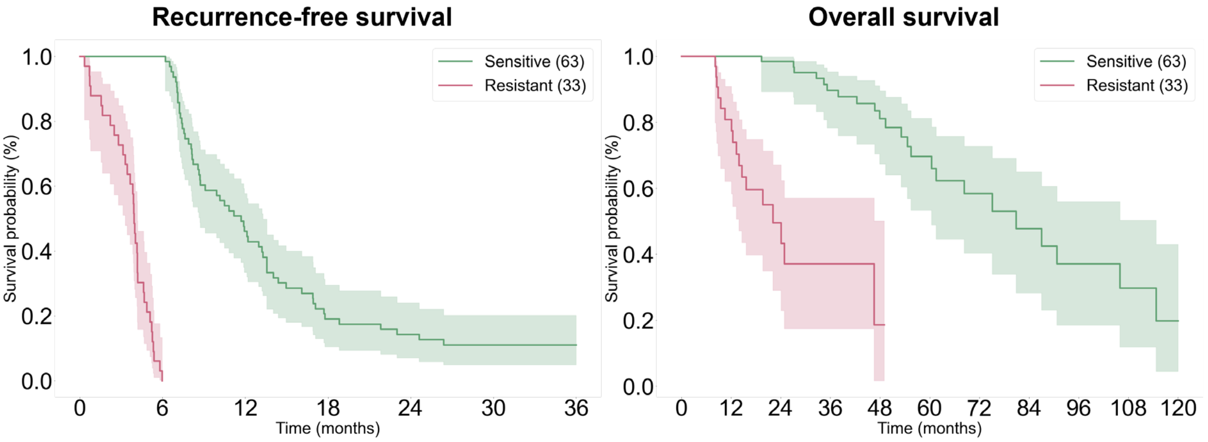

Supplementary Figure 2. Importance of features in the combined model over 5 folds.

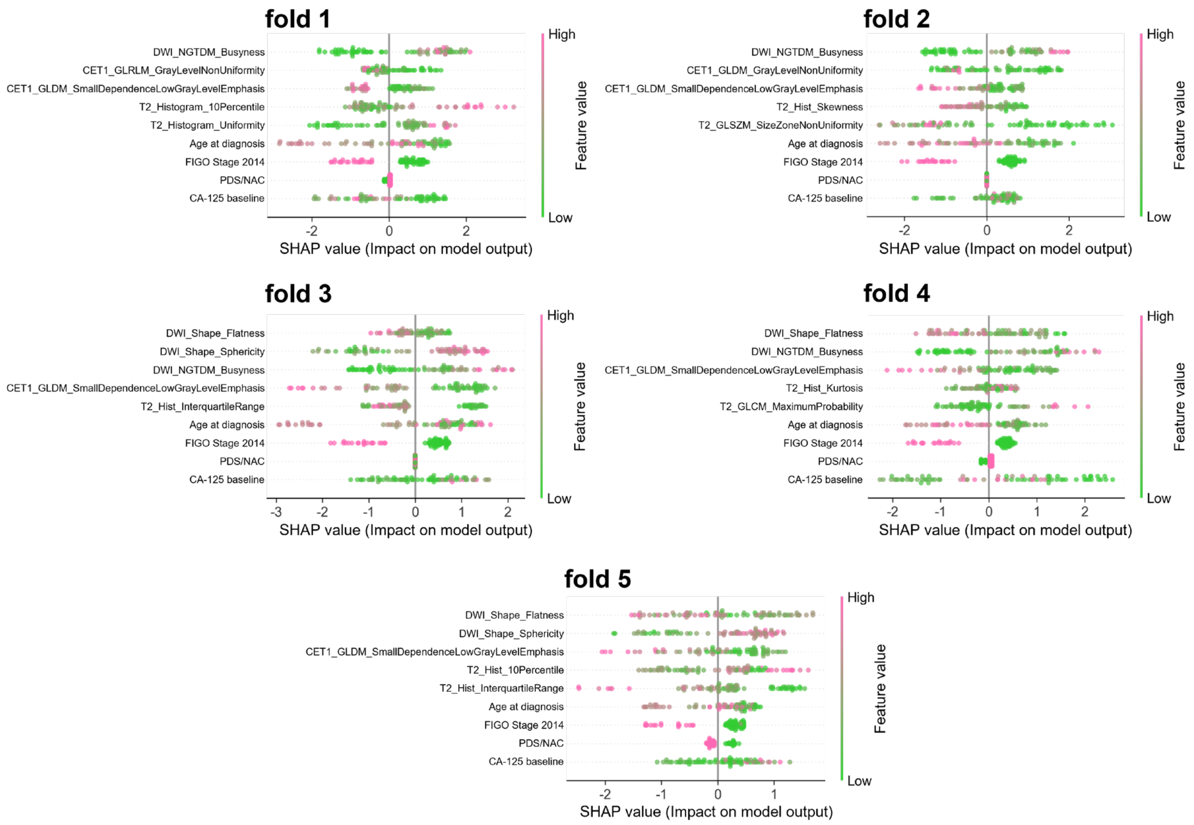

Supplementary Table S1. Extracted 107 radiomics features

**Shape**			
Elongation	Flatness	Least Axis Length	Major Axis Length
Maximum 2D Diameter Column	Maximum 2D Diameter Row	Maximum 2D Diameter Slice	Maximum 3D Diameter
Mesh Volume	Minor Axis Length	Sphericity	Surface Area
Surface Volume Ratio	Voxel Volume		

**Histogram**			
10 Percentile	90 Percentile	Energy	Entropy
Interquartile Range	Kurtosis	Maximum	Mean Absolute Deviation
Mean	Median	Minimum	Range
Robust Mean Absolute Deviation	Root Mean Squared	Skewness	Total Energy
Uniformity	Variance		

**Texture GLCM**			
Autocorrelation	Cluster Prominence	Cluster Shade	Cluster Tendency
Contrast	Correlation	Difference Average	Difference Entropy
Difference Variance	Id	Idm	Idmn
Idn	Imc1	Imc2	Inverse Variance
Joint Average	Joint Energy	Joint Entropy	MCC
Maximum Probability	Sum Average	Sum Entropy	Sum Squares

**Texture GLSZM**			
Gray Level Non Uniformity	Gray Level Non Uniformity Normalized	Gray Level Variance	High Gray Level Zone Emphasis
Large Area Emphasis	Large Area High Gray Level Emphasis	Large Area Low Gray Level Emphasis	Low Gray Level Zone Emphasis
Size Zone Non Uniformity	Size Zone Non Uniformity Normalized	Small Area Emphasis	Small Area High Gray Level Emphasis
Small Area Low Gray Level Emphasis	Zone Entropy	Zone Percentage	Zone Variance

Texture GLRLM			
Gray Level Non Uniformity	Gray Level Non Uniformity Normalized	Gray Level Variance	High Gray Level Run Emphasis
Long Run Emphasis	Long Run High Gray Level Emphasis	Long Run Low Gray Level Emphasis	Low Gray Level Run Emphasis
Run Entropy	Run Length Non Uniformity	Run Length Non Uniformity Normalized	Run Percentage
Run Variance	Short Run Emphasis	Short Run High Gray Level Emphasis	Short Run Low Gray Level Emphasis

Texture NGTDM			
Busyness	Coarseness	Complexity	Contrast
Strength			

Texture GLDM			
Dependence Entropy	Dependence Non Uniformity	Dependence Non Uniformity Normalized	Dependence Variance
Gray Level Non Uniformity	Gray Level Variance	High Gray Level Emphasis	Large Dependence Emphasis
Large Dependence High Gray Level Emphasis	Large Dependence Low Gray Level Emphasis	Low Gray Level Emphasis	Small Dependence Emphasis
Small Dependence High Gray Level Emphasis	Small Dependence Low Gray Level Emphasis		
